# A quest for quality care: Exploration of a model of leadership relationships, work engagement, and patient outcomes

**DOI:** 10.1111/jan.14583

**Published:** 2020-10-12

**Authors:** Jenny M. Parr, Stephen Teo, Jane Koziol‐McLain

**Affiliations:** ^1^ Patient and Whaanau Experience Counties Manukau Health District Health Board Middlemore Hospital Auckland New Zealand; ^2^ Business and Law School of Business and Law Edith Cowan University Joondalup WA Australia; ^3^ Centre for Interdisciplinary Trauma Research Auckland University of Technology Northcote Auckland New Zealand

**Keywords:** patient outcomes, patient satisfaction, resonant leadership, social exchange theory, work engagement

## Abstract

**Aim:**

To explore the effects of resonant leadership, leader exchange relationships and perceived organizational support on work engagement and patient outcomes.

**Design:**

A cross‐sectional survey design.

**Methods:**

Data were collected in June and July 2016 from 252 nurses and clerical staff and institutional patient safety (falls rates) and patient satisfaction (Friends and Family Test) in New Zealand. Data were analysed with structural equation modelling (SEM).

**Results:**

The final model was an excellent fit to the data (χ^2^ (22, *N* = 252) = 39.048, *p* = 0.014). Resonant leadership was significantly and positively associated with relationships at work, perception of unit care quality (β = 0.28, *p* < 0.001), reduced falls rates (β = −0.14, *p* < 0.05) and better patient satisfaction (β = −0.41, *p* < 0.001). A direct effect of resonant leadership was demonstrated on patient satisfaction (β = 0.20, *p* < 0.01). Perceived organization support (β = 0.40, *p* < 0.001) and leader–member exchange (β = 0.46, *p* < 0.001) were confirmed antecedents of work engagement. Work engagement was confirmed as an antecedent of nurse perception of unit care quality (β = 0.21, *p* < 0.001). Where social exchanges exist, work engagement mediates these. Three further mediated paths bypassed work engagement altogether.

**Conclusion:**

Existing literature investigating the drivers and impacts of work engagement predominantly focuses on staff outcomes rather than patient outcomes. The findings identify modifiable factors to improve staff experience, patient safety, and ultimately patient satisfaction. Resonant leadership, a relational style, is a core antecedent of quality care and positively associated with staff experience and patient outcomes.

**Impact:**

This investigation into a real‐world problem for nurse leaders also confirmed that an organizational focus on work engagement is not always required. Resonant leadership improves staff work experience, patient safety, and patient satisfaction. Nurse leaders should measure, foster, and develop resonant leadership in practice.

## 摘要

**目标:**

探讨共鸣式领导、领导交流关系和组织支持认知对工作投入和患者结果的影响。

**设计:**

横断面调查设计。

**方法:**

在2016年6月至7月从新西兰252名护士和文书人员以及机构患者安全(跌倒率)和患者满意度(亲友测试)中采集数据。使用结构方程建模(SEM)对数据进行分析。

**结果:**

最终模型与数据拟合良好(χ^2^ (22, *N* = 252) = 39.048, *p* = 0.014)。共鸣式领导与工作关系、对单位护理质量的认知(β=0.28,*p* < 0.001)具有显著地积极作用,并降低跌倒率(β=‐0.14,*p* < 0.05)以及提升患者的满意度(β=‐0.41,*p* < 0.001)。共鸣式领导对患者满意度具有直接影响作用(β = 0.20, *p* < 0.01)。组织支持认知(β = 0.40, *p* < 0.001)和领导‐成员之间的交流(β = 0.46, *p* < 0.001)是工作投入的前因变量。同时,工作投入是护士对单位护理质量认知的前因变量(β = 0.21, *p* < 0.001)。在存在社会交流的场所中,工作参与起到中介作用。另外三个中介因素与工作参与不相关。

**结论:**

现有的文献调查了工作投入的驱动因素和影响,侧重于员工的结果而非患者的结果。研究发现确定了可改善员工体验、提高患者安全性并最终提升患者满意度的可变因素。共鸣式领导是一种关系型领导方式,是优质护理的核心前因变量,对员工体验和患者结果具有积极的影响作用。

**影响:**

这项对于护士领导者实际问题的调查也证实组织对工作投入的关注并非绝对必要。共鸣式领导能改善员工的工作体验、提高患者安全性和提升患者满意度。护士领导者应在实践中衡量、培养和发展他们的共鸣式领导能力。

## 1 INTRODUCTION

Nurse executives globally are expected to articulate the contribution of nursing to patient care within the boardroom (Mastal et al., 2007). This is becoming more important as healthcare organizations are under pressure to control costs (Francis Inquiry, 2013; Needleman, 2016). Nursing leadership is often held to account for the quality of patient care (Department of Health, 2014; Francis Inquiry, 2013; Healthcare Commission, 2006, 2007, 2009) despite an absence of research‐relating nursing leadership to nurse sensitive outcome indicators. However, notwithstanding over 20 years of discourse about measuring the contribution of nursing to patient care and its importance (Aiken et al., 2014; Ausserhofer et al., 2014), there remains a lack of consensus on metrics (Dubois et al., 2013) and no single measure of ward‐level quality care (Dubois et al., 2013; Hurst, 2011; Parr et al., 2018). Nurse executives continue to be challenged with insufficient evidence to guide decisions on how to organize and lead nursing to affect gains in patient safety, clinical effectiveness and patient experience.

Evidence is emerging which supports the view that relational nursing leadership has a positive relationship with patient outcomes (Squires, 2010; Wong et al., 2013). The implication, therefore, is that nursing leadership should be a focus for organizations intent on improving patient outcomes (Wong et al., 2013). Nursing work is highly relational, where staff need to connect with patients as they provide physical and psychosocial care (Feo et al., 2017). Critical relational components of nursing practice such as engaging with patients, being present with them, and helping them to cope (Feo et al., 2017) are highly emotional and require relational energy (Cummings, 2004). It also requires staff to be positive, fulfilled (Schaufeli et al., 2006), and willing, and able to reciprocate perceived support from employers and managers with discretionary effort (Eisenberger et al., 1997) to connect in this way. How these characteristics of nursing interact in the complex healthcare setting, however, is not well understood. Our research aim was to test a model linking resonant leadership with experiences of leader–member exchange relationships, perceived organization support, work engagement, perception of unit care quality, patient safety, and patient satisfaction.

### 1.1 Theoretical framework

Social Exchange Theory provides a relational frame to consider patient experience and the reciprocal nature of engagement between staff and patients and families (Saks, 2006). That is, interactions among patients, family, and staff lead to obligations, which are interdependent and contingent on each other and may be of high or low quality (Cropanzano & Mitchell, 2005). As patient experience is effectively relational, there is a strong fit with considering these measures within research with Social Exchange Theory as the theoretical basis.

Within Social Exchange Theory, interactions lead to obligations which are interdependent and contingent on one another, with the potential to develop high‐quality relationships (Cropanzano & Mitchell, 2005). The ‘exchange’ is bi‐directional between two parties and includes (a) rules and norms of exchange, (b) resources exchanged, and (c) emerging relationships (Cropanzano & Mitchell, 2005, p. 875). Interdependence is characterized by ‘mutual and complementary arrangements’ (Cropanzano & Mitchell, 2005, p. 876). By obeying rules over time, relationships evolve into trusting, loyal, and mutual commitments. Rules of exchange may involve reciprocity or negotiation. Reciprocity is not explicitly negotiated, but understood and contingent on behaviour, may reflect cultural expectations such as expected behaviour or a norm/individual orientation. Reciprocal exchanges generate better work relationships than negotiated relationships, permitting more trust of and commitment to each other.

Cropanzano and Mitchell (2005) described a model for the relationship between perceived organizational support and the Leader–Member Exchange or the quality of the relationship. Within this, it is important to consider all the domains of leadership which include the leader, the follower, and the relationship (Graen & Uhl‐Bien, 1995). Social Exchange Theory recognizes the importance of the quality of the relationship between the leader and member as the basis of the social exchange as individuals return benefits they receive and are likely to match these to the person with whom they have a social exchange relationship (Cropanzano & Mitchell, 2005). Practice environment aspects are also considered within Social Exchange Theory, in relation to Perceived Organization Support, or the degree to which the employee perceives the organization cares about their well‐being and values their contribution (Eisenberger et al., 1997). An employee who perceives their employer is supportive is more likely to reciprocate.

Social exchanges are a fundamental mechanism in the interplay between leadership and engagement. The quality of the leader–nurse relationship is evidenced to be predicted by resonant leadership (Squires et al., 2010). The individual roles that the quality of the relationship with the organization and the quality of the relationship between the leader and the nurse play as antecedents of engagement (Brunetto et al., 2014; Dasgupta, 2016; Shacklock et al., 2013) and nurse perceived quality of care (Van Bogaert et al., 2012, 2013; Wong et al., 2010) have also been highlighted. Social Exchange Theory has been demonstrated as a useful perspective when investigating work relationships (Brunetto et al., 2014; Dasgupta, 2016; Saks, 2006; Shacklock et al., 2013; Squires et al., 2010; Trinchero et al., 2013). What is not evident is the importance of these constructs in relation to leadership as an antecedent and the relationships with work engagement and patient outcomes as dependent variables.

**Figure 1:**
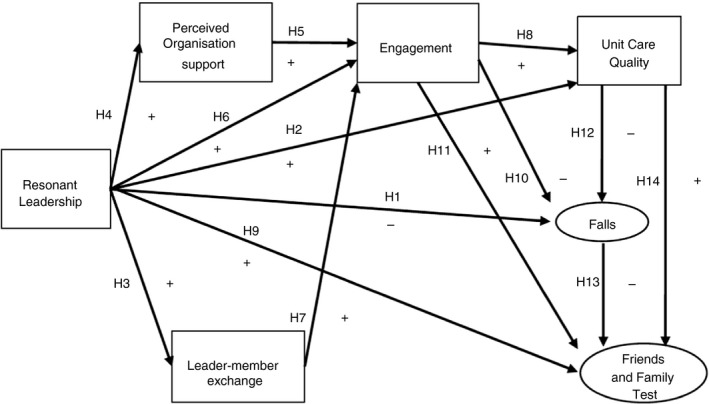
Hypothesised a priori model

With a Social Exchange lens, we focus on the constructs of relational leadership, perceived organization support, leader–member exchange, nurse engagement and patient outcomes. The study constructs and hypothesized model (Figure 1) are reviewed in the following section.

## 2 BACKGROUND

### 2.1 Resonant leadership

Relational leadership styles which focus on people and relationships to achieve the common goal are now favoured over task‐oriented styles (Cummings, et al., 2010). Resonant leadership styles are described as visionary, coaching, affiliative and democratic (Cummings et al., 2005). Resonant leaders are those in tune with the people around them, they know and can communicate what to do and why to do it and have a high level of emotional intelligence (McKee & Massimilian, 2006, p. 45).

The relational leader appears to have a positive effect on relationships, safety culture and perception of exposure to adverse events such as medication errors (Wong et al., 2013). Safety climate was affected by leader–member relationships and the work environment and a small effect was seen on nurse‐reported medication errors (*r* = −0.22; Squires et al., 2010). Cummings et al. (2010) demonstrated that high‐resonant leadership styles were significantly associated with 26% lower odds of mortality. The nurse management at the unit level is associated with nurse perception of quality care (*R* ^2^ = 0.61, *p* < 0.05; Van Bogaert et al., 2009). Vogus and Sutcliffe (2007) demonstrated that a combination of high ‘trust in the manager’ and high ‘use of care pathways’ is related to lower numbers of reported medication incidents. However, these patient safety outcomes were primarily nurse reported and subject to common method bias. Purdy et al. (2010) showed that fewer falls per 1,000 bed days were predicted when empowering workplaces had positive effects on nurse‐assessed quality of care. This research aimed to use data that reflected the contribution of nurses to quality care (Dubois et al., 2013) and are already collected and available. These studies led to the following hypotheses:

H 1 There is a negative relationship between resonant leadership and falls.

H 2 There is a positive relationship between resonant leadership and perceptions of unit care quality.

### 2.2 Leader–member exchange (LMX)

LMX focuses on the two‐way (dyadic) relationship between the leader and subordinate rather than the personal characteristics of the leader, the situation, or the interplay (Gerstner & Day, 1997). The concept of reciprocity is, therefore, a fundamental component. Three domains make up this theory – the leader, the follower, and the relationship, with the emphasis on all three in combination (Graen & Uhl‐Bien, 1995). Measurement of the quality of the leader–member relationship, such as the Charge Nurse Manager and registered nurses, has demonstrated that resonant leadership is associated with the quality of the relationship (correlation coefficient 0.52, pathways significant at *p* < 0.05; Squires et al., 2010). This led to the following hypothesis:

H 3 There is a positive relationship between resonant leadership and exchange relationships.

### 2.3 Perceived organization support

Given the emotional nature of nursing work and the requirement to provide effort beyond the bounds of the employment contract, Perceived Organization Support becomes important. The voluntary nature of discretionary donation of resources is considered to be more highly valued than if it was not voluntary and benefits received in return are likely to be greater (Eisenberger et al., 1997). Perceived Organization Support, therefore, reflects ‘the extent to which the organization values their contribution and cares about their wellbeing and provides a basis for deciding whether increased effort for the organization will be noticed and rewarded’ (Eisenberger et al., 1997, p. 818). Although no existing literature was identified demonstrating the relationship between resonant leadership and perceived organizational support, Squires et al. (2010) used the Perceived Nursing Work Environment PNWE of Critical Care Nurses (Choi et al., 2004) and revealed large effect sizes. It is, therefore, theoretically plausible to explore these relationships. This led to the following hypothesis:

H 4 There is a positive relationship between resonant leadership and perceived organizational support.

### 2.4 Work engagement

Work engagement is defined as ‘a positive, fulfilling, work‐related state of mind that is characterized by vigor, dedication and absorption… a persistent and pervasive affective–cognitive state that is not focused on any particular object, event, individual, or behaviour’ (Schaufeli & Bakker, 2004, p. 295). Saks (2006) demonstrated the reciprocal element of organizational support and work engagement, suggesting that there is more likelihood of trusting and high‐quality relationships with their supervisor where staff are more engaged. There is also support for work engagement being predicted by exchange relationships (t‐statistic = 2.57, significant at *p* < 0.01; Shacklock et al., 2013). The quality of the relationship between the supervisor and the member and their perception of organizational support predict work engagement and employees more satisfied with the relationship have higher levels of work engagement (Brunetto et al., 2014; Dasgupta, 2016; Shacklock et al., 2013). These studies led to the following hypotheses:

H 5 There is a positive relationship between perceived organisational support and work engagement.

H 6 There is a positive relationship between resonant leadership and work engagement.

H 7 There is a positive relationship between exchange relationships and work engagement.

### 2.5 Quality of care and patient outcomes

Quality is ‘the degree to which a system of production meets (or exceeds) the needs and desires of the people it serves’ (Berwick, 2013, p. 11) and comprises three domains: safety, patient experience, and effectiveness. Falls is used as a measure of patient safety in the literature (Duffield et al., 2011). Patient experience comprises several components: patient satisfaction, patient perception, patient engagement, patient participation, and patient preferences (LaVela & Gallan, 2014). Patient satisfaction reflects the patient's end‐state judgment of achieved objectives (LaVela & Gallan, 2014). Falls and measures of patient satisfaction are widely acknowledged to be examples of nurse‐sensitive outcome indicators as they detect changes in a patient's condition (Dubois et al., 2013). Although the literature confirms the use of quality patient outcome indicators (He et al., 2016), the use of patient experience data and readily available institutional data gathered through the process of care delivery and evaluation is limited.

The relationship between work engagement and nurse perception of unit care quality has been demonstrated (Van Bogaert et al., 2012). Research has also demonstrated that the quality of the exchange (Perceived Organization Support) is related to organizational commitment and turnover intentions, while the quality of the relationship (Leader–Member Exchange) as the basis of the exchange has predicted job satisfaction and performance. These are important constructs that explain the nature of reciprocity, predict work engagement and are relevant in the nursing context. The interdependent nature of social exchanges may help to explain a relationship between resonant leadership and nurse perception of unit care quality, patient safety, and patient satisfaction. Leader–member interactions may lead to obligations to reciprocate by adopting a local folk belief about the quality of care, exchanging nursing services, and building relationships with patients as mutual investment develops (Cropanzano & Mitchell, 2005). Therefore, we proposed the following hypotheses:

H 8 There is a positive relationship between level of work engagement and perceptions of unit care quality.

H 9 There is a positive relationship between resonant leadership and Friends and Family Test.

H 10 There is a negative relationship between level of work engagement and falls.

H 11 There is a positive relationship between level of work engagement and Friends and Family Test.

Nurse‐reported perceptions of unit care quality (Lake, 2002) is often used to understand quality of care. This may be due to the significant challenges of evaluating nursing care due to the laborious nature of identifying and measuring nurse‐sensitive measures which persist decades after Donabedian highlighted them (Parr et al., 2018). A significant correlation was found between nurse perception of unit care quality and nurse‐reported falls and patient satisfaction (Purdy et al., 2010). Although no existing literature was identified to demonstrate relationships between falls and the Friends and Family Test and perception of unit care quality and Friends and Family Test, the obligations and mutual investment generated within these social exchanges led to the following hypotheses:

H 12 There is a negative relationship between perceptions of unit care quality and falls.

H 13 There is a negative relationship between falls and Friends and Family Test.

H 14 There is a positive relationship between perceptions of unit care quality and Friends and Family Test.

### 2.6 Hypothesized model

Resonant leadership is evidenced as an antecedent to the quality of the leader–nurse relationship (Squires et al., 2010). The work environment has been investigated in the context of patient outcomes but not in research involving leadership styles. What is also not evident is the importance of these constructs in relation to leadership as an antecedent and the relationships with work engagement and patient outcomes as dependent variables. The purpose of this study was to test a model linking resonant leadership with experiences of leader–member exchange relationships, perceived organization support, work engagement, nurse perception of unit care quality, patient safety, and patient satisfaction. Therefore, we proposed a serial mediation hypothesis (Figure 1):

H 15 that work engagement mediates the positive relationship between resonant leadership, exchange relationships, organisational support, unit care quality the negative association with falls and Friends and Family Test.

## 3 THE STUDY

### 3.1 Aim

The aim of this study was to explore the effects of resonant leadership, leader/member exchange relationships and perceived organizational support on work engagement and unit‐level patient outcomes.

### 3.2 Design

Data from a cross‐sectional self‐report survey of nurses and clerical staff called the Leadership and Engagement of Nurses (LEON) survey and institutionally collected patient safety (falls rates) and patient satisfaction (Friends and Family Test) data were analysed using structural equation modelling (SEM). SEM models the relationships among multiple independent and dependent constructs and simultaneously allows researchers to answer a set of interrelated research questions in a single, systematic, and comprehensive analysis contrary to first‐generation statistical tools such as regression (Anderson & Gerbing, 1988). This approach uses a measurement model specified a priori to assess and confirm convergent and discriminant validity and a structural model to undertake a confirmatory assessment of nomological validity (Anderson & Gerbing, 1988).

### 3.3 Participants

The participants, 252 registered nurses, enrolled nurses, and healthcare assistants, as well as administrative and clerical staff, worked in 1 of 20 units across adult inpatient medical surgical wards at two hospital sites in urban New Zealand. These staff were all managed by their unit manager and considered to contribute to the unit's quality outcomes. The inclusion of clerical staff is consistent with the approach taken by White, Wells and Butterworth (2014) who considered that all team members contribute to the quality of care on the ward.

Considering the complexity or size of the model, a sample size of 10–20 cases per included measured variable is appropriate (Bentler & Chou, 1987; Lomax & Schumacker, 2004). As this research had eight variables, a sample of 200 was acceptable (Squires, 2010).

### 3.4 Data collection

#### 3.4.1 Survey

Data were collected over 2 months, June ‐July 2016. An information sheet explaining the research, voluntarily participation, and contact details of the researchers in case of questions was provided to all eligible staff. Participants were asked to complete the online survey, with an option to complete a paper survey and return in the internal post. An independent person using the work email system and the LEON email address contacted participants. A poster was displayed, and reminders were sent to units to remind staff that the research was still seeking participants and to highlight the remaining time for completion at handover and ward meetings. This approach, recommended by Dillman (2000) and Babbie (2013), was repeated during the 2 months of collection.

#### 3.4.2 Institutional data

The falls and Friends and Family Test data were routinely collected by the institution in the process of service delivery and service improvement and were also collected for the period of June‐July 2016.

#### 3.4.3 Measurements

The study was comprised of eight variables; four independent variables, three dependent variables, and one marker variable. Table 1 describes the variables, constructs, and psychometric properties of the LEON survey scales (Table 1).

**Table 1:** Variables, constructs, and psychometric properties of LEON survey scales

Variable	Construct	Scale/measure	Measurement	Scoring	Reliability	Validity
Independent	1. Resonant leadership	Resonant leadership scale (Estabrooks et al., 2009)	10 items measuring components of resonant leadership	Likert scale (1–5) for each item. Means of those who answered (1)– (5) used as resonant leadership score	High internal consistency for total scale α = 0.95	Face/content validity Correlations between variables above 0.5, most above 0.6.
Independent	2. Leader–member exchange	Leader–member Exchange (LMX‐7) Graen and Uhl‐Bien (1995)	7 items measuring the satisfaction of employees with their relationship with their supervisor	Likert scale (1–5) for each item	Internal consistency from the member's perspective (α = 0.89)	Reported to have predictive validity
Independent	3. Perceived Organisation Support	Perception of Organisational Support (POS) (Eisenberger et al., 1997)	8 items measuring perception of organizational support	Likert scale (7–1) for each item	High internal reliability α = 0.90 and goodness of fit α = 0.94	Reported to have discriminant validity
Independent	4. Work Engagement	Utrecht Work Engagement Scale (UWES) (Schaufeli et al., 2006)	9 items measuring three factors of work engagement; vigour, dedication, and absorption	Likert scale (0–6) for each item	High internal reliability α = 0.89–0.97	Factorial validity variances between countries for and internal consistency (α = 0.60 to 0.88, respectively, median = 0.77).
Dependant	5. Perception of unit care quality	Perceptions of unit care quality Aiken et al. (2001) and Aiken et al. (2002)	4 items measuring perceptions of care on their unit	Likert scale (1–4) for 3 items and 1–3 for 1 item		Not reported
Marker	6. Willingness to try new food products DSI	Willingness to try new food products; DSI scale Goldsmith and Hofacker (1991) adapted by Barcellos et al. (2009)	6 items	Likert scale (1–5) for each item	Good reliability in Brazil (α = 0.80) and in the UK (α = 0.78).	Reported to have predictive validity
Dependant	7. Patient safety	Falls (Purdy et al., 2010)	Number of falls recorded by the institution	Number per 1,000 bed days		
Dependant	8. Patient satisfaction	Friends and Family Test (Department of Health, 2013).	1 item measuring likelihood to recommend for similar care or treatment	Percentage of promoters (score 5) over detractors (scores 1 & 2) across a 5‐point scale.		

#### 3.4.4 Independent variables

##### Resonant leadership

Resonant leadership was measured using the 10‐item Resonant Leadership Scale which is a subscale of the Alberta Context Tool (Cummings, 2004; Cummings et al., 2008; Estabrooks et al., 2009). Participants were asked to rate the extent to which their immediate supervisor displays leadership behaviours using a 5‐point Likert‐type scale from ‘strongly disagree’ (1) to ‘strongly agree’ (5). A sample statement is ‘the leader in my clinical program or unit acts on values even if it is at a personal cost’.

##### Perceived organization support

Perceived Organization Support was measured using the 8‐item Perceived Organization Support scale (Eisenberger et al., 1997). Participants were asked to indicate the extent of their agreement with each item on a 7‐point Likert‐type scale from ‘strongly agree’ (1) – ‘strongly disagree’ (7). A sample question is ‘My organisation cares about my opinions’.

##### Leader–member exchange

The validated 7‐item Leader–Member Exchange (LMX‐7) scale developed by Graen and Uhl‐Bien (1995) was used to measure the satisfaction of employees with their relationship with their leader. Participants respond on a 5‐point scale ranging from ‘to a very little extent’ (1) to ‘to a very great extent’ (5). A sample statement is ‘How effective would you characterize your working relationship with your supervisor?’

##### Work engagement

Work engagement was measured using the shortened form of the Utrecht Work Engagement Scale. Participants were asked to answer statements about how they feel at work on a scale of ‘never’ (0) to ‘always/every day’ (6). A sample statement is ‘at my work I feel bursting with energy’.

#### 3.4.5 Dependent variables

##### The perception of unit care quality

The perception of unit care quality was measured using a 4‐item short scale originally used by Aiken et al. (2002). A sample question is ‘In general, how would you describe the quality of nursing care delivered to patients on your unit?’ (excellent, good, fair, or poor).

##### Patient safety

Falls is the proxy measure for patient safety and is measured by the number of falls recorded by the institution reported as the number per 1,000 bed days (Purdy et al., 2010).

##### Patient satisfaction

The Friends and Family Test is the proxy measure for patient satisfaction. The Friends and Family Test asks the question ‘How likely are you to recommend our ward to friends and family if they needed similar care or treatment?’ (Department of Health, 2013). It is reported as a percentage of promoters (score 5) over detractors (score 1 & 2) across a 5‐point scale. Single‐item global measures can allow respondents to consider all aspects of a phenomenon (Patrician, 2004).

##### Marker variable

Common method bias is a concern when combining multiple self‐report variables into independent and dependent variables (Podsakoff et al., 2003). To avoid potentially misleading findings, a ‘marker variable’ is suggested by Podsakoff et al. (2003) to be used as a statistical remedy for common method bias. The marker variable must be theoretically unrelated to one or all of the constructs in the research (Podsakoff et al., 2003). We selected the willingness to try new food products DSI scale (Barcellos et al., 2009) as a ‘marker variable’ (social desirability scale). An example of an item in this scale was ‘I buy new, different or innovative foods before anyone else I know’.

### 3.5 Ethical considerations

Approval for the study was obtained from the Auckland University of Technology Ethics Committee (19 April 2016) and locality approval was granted from the organization involved in the study (January 2016).

### 3.6 Data analysis

Data analysis was conducted using IBM SPSS Statistics 25.0® software and IBM AMOS 25.0® software for structural equation modelling. Confirmatory factor analysis using the two‐step approach suggested by Anderson and Gerbing (1988) was employed to test the significance of the scales as the instruments were being used in New Zealand for the first time (Hinkin et al., 1997). One factor congeneric models were reviewed for goodness of fit using the chi‐squared statistic of goodness‐of‐fit cut‐off criteria recommended by Hu and Bentler (1999). The structural equation model was tested with the data. Path coefficients are interpreted as suggested by Cohen: absolute values from 0.10 to 0.30 are considered small, 0.30–0.50 medium, and 0.50 and above large (Cohen, 1992). Finally, path and mediation analysis was conducted using PROCESS v2.16.3 in IBM SPSS Statistics 25.0 (Hayes, 2013) with a 95% confidence interval based on 10,000 bootstrap samples.

### 3.7 Validity reliability and rigour

The seven steps outlined in Hinkin et al. (1997) were followed to ensure the measures used in the LEON survey were valid and reliable. All variables of interest, measures, number of items retained in the final model, means, standard deviations, alphas, and score ranges are described in Table 2. The measurement model was tested for discriminant validity, demonstrated (AVE > 0.5) convergent validity and fit to the data (Hu & Bentler, 1999). Tests for common method bias suggested by Podsakoff et al. (2003) were undertaken. The psychometric properties of the variables of interest are presented in Table 2.

**Table 2:** Descriptive statistics, average variance estimates, composite reliability coefficients, and inter‐correlations for the study variables

Scale/item	Mean	*SD*	Range	Score range	Items	α	AVE	1	2	3	4	5	6	7	8
1. Resonant Leadership (RL)	3.729	0.765	1–5	4.00	5	0.88	0.597								
2. Leader–Member Exchange (LMX)	3.605	0.905	1–7	4.00	3	0.80	0.592	0.759**							
3. Perceived Organization Support (POS)	4.478	1.362	1–7	6.00	5	0.91	0.690	0.461**	0.430**						
4. Work Engagement (ENG)	4.810	0.939	0–6	4.75	4	0.83	0.584	0.284**	0.370**	0.457**					
5. Perception of unit care quality (PUCQ)^a^	0.027	0.844	‐	3.41	2	0.74	0.656	0.324**	0.303**	0.291**	0.303**				
6. Willingness to Try New Food Products DSI	2.777	0.866	1–5	4.00	6	0.94	‐	−0.105	−0.082	−0.127*	−0.049	−0.077			
7. Fall (FALL)	94.041	4.777	0–100	20.19	1	‐	‐	0.116	0.117	0.089	−0.023	0.179**	−0.078		
8. Friends and Family Test (FFT)	76.603	15.009	0–100	56.00	1	‐	‐	0.217**	0.103	−0.009	−0.096	0.087	−0.068	0.420**	‐

Note

*N* = 252.

Abbreviations: AVE, average variance extracted; Items = number of items retained in the final model;*SD*, standard deviation.

^a^ Standardized.

* *p* < 0.05,

** *p* < 0.01,

*** *p* < 0.001.

## 4 RESULTS

A final sample of 252 completed and usable LEON survey responses were obtained (response rate = 26.4%) following missing value analysis (χ^2^ = 169.659, *df* = 198, Sig. = 0.928). Units with no institutional data, cases where the unit were not specified, and influentials were removed (*n* = 213). Most participants were Registered Nurses (73%), female (86.5%), worked full time (60%) and were under 35 (44.4%) (Table 3). Twenty‐five per cent had been in practice 3 years; a small proportion of registered nurses and enrolled nurses (*N* = 15, 7.9%) were in their first year of practice.

**Table 3:** Observed frequencies, means, and standard deviations for LEON survey respondent's demographic characteristics and demographics (*N* = 252)

Demographic characteristics	*N* (%)
Gender	
Female	218 (86.5)
Male	32 (12.7)
Transgender	2 (0.8)
Age	
24 and under	26 (10.3)
25–34	86 (34.1)
35–44	49 (19.4)
45–54	52 (20.6)
55–64	33 (13.1)
65 and over	6 (2.4)
Role	
Charge nurse manager	17 (6.7)
Registered nurse (including ACCN)	184 (73.0)
Enrolled nurse	7 (2.8)
Health care assistant	24 (9.5)
Ward clerk, administrative assistant, or admin clerk	20 (7.9)
Highest education	
High school	33 (13.1)
Vocational certificate	15 (6.0)
Baccalaureate degree	103 (40.9)
Post‐graduate certificate	49 (19.4)
Post‐graduate diploma	38 (15.1)
Master's degree	13 (5.2)
Unit speciality	
Medical or surgical	235 (93.3)
Assessment or short stay	15 (6.0)
Mental health, post‐acute, or critical care	2 (0.8)
Employment status	
Full‐time	152 (60.3)
Part‐time	100 (39.7)

Demographics	*N*	Mean	*SD*
Years in professional practice	251	12.15	11.23
Years on current unit	252	4.65	5.15
Years at current organization	252	6.88	7.09

Nurses reported the leadership of their managers to be highly resonant (mean 3.73, *SD* = 0.77); this was higher than Canadian studies from Spence Laschinger et al. (2014) (mean 3.22, *SD* = 0.94) and Bawafaa et al. (2015) (mean 3.23, *SD* = 0.94) where the sample sizes were both greater than 1,200. Overall, staff reported work engagement the highest (mean 4.81, *SD* = 0.94), leader–member exchange relationships the lowest (mean 3.61, *SD* = 0.91), and perceived organization support to be moderate (mean 4.48, *SD* = 1.36). The measurement model had discriminant and convergent validity and excellent fit (χ^2^ (141, *N* = 252) = 175.834, TLI = 0.984, CFI = 0.987, CMIN/DF = 1.247, RMSEA = 0.031, SRMR = 0.0415, PCLOSE = 0.988). The difference of correlations of all constructs between, before, and after including the marker variable was acceptable at less than 0.2 (0.045) (Lindell & Whitney, 2001).

### 4.1 Hypothesis testing

**Figure 2:**
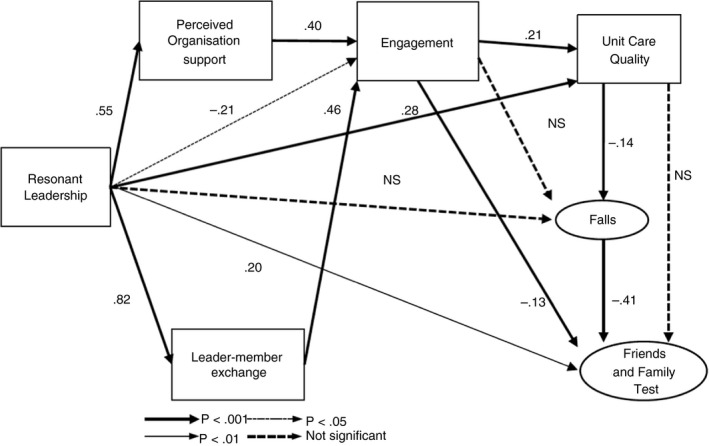
Final model paths and standardised effect estimates

The initial path model demonstrated a very good fit (χ^2^ (19, *N* = 252) = 34.019, TLI = 0.954, CFI = 0.976, CMIN/DF = 1.790, RMSEA = 0.056, SRMR = 0.0377, PCLOSE = 0.339). Paths that were not significant were deleted (H1, H10, and H14). There were no positive modification indices to address. With these modifications, the path model demonstrated an excellent fit to the data (χ^2^ (22, *N* = 252) = 39.048, TLI = 0.955, CFI = 0.973, CMIN/DF = 1.775, RMSEA = 0.056, SRMR = 0.0418, PCLOSE = 0.344; Figure 2).

The final model demonstrated partial support for the a priori model (Figure 1). Higher resonant leadership was associated with both positive exchange relationships (H3) and a positive perceived organizational support (H4). Positive exchange relationships were associated with higher levels of work engagement (H7), as was a positive perceived organizational support (H5). Higher resonant leadership was associated with higher perceptions of unit care quality (H2) and better patient experience (measured by the Friends and Family Test) (H9). However, higher resonant leadership was associated with a lower level of work engagement (H6) (small effect; β = −0.21, *p* < 0.05) and higher work engagement was associated with worse patient experience (H11) (small effect; β = −0.13, *p* < 0.05) which were unexpected. Higher levels of nurse perception of unit care quality were associated with lower rates of falls (H12) (β = −0.14, *p* < 0.05) which were associated with better patient experience (H13) (β = −0.41, *p* < 0.001).

### 4.2 Effect estimates

The final model revealed large effect sizes for the positive relationships between resonant leadership and perceived organization support (H4) (β = 0.55, *p* < 0.001) and resonant leadership and leader–member exchange (H3) (β = 0.82, *p* < 0.001) (Figure 2 and Table 4a). Medium effects were found for the positive relationship between perceived organization support and work engagement (H5) (β = 0.40, *p* < 0.001), the positive relationship between leader–member exchange and work engagement (H7) (β = 0.46, *p* < 0.001), and the negative relationship between falls and the Friends and Family Test (H13) (β = −0.41, *p* < 0.001). All other effects (both positive and negative) were small (Table 4).

**Table 4:** Direct and indirect effect estimates. (a) Direct effect estimates. (b) Indirect effect of Resonant Leadership on Friends and Family Test through POS, QUAL, and FALLS

(a) Structural paths	Unstandardized estimate	*p*
H2: Resonant leadership → Perception of Unit Care Quality	0.411	0.000***
H3: Resonant leadership → Leader–Member Exchange	1.153	0.000***
H4: Resonant leadership → Perceived Organization Support	1.249	0.000***
H5: Perceived Organization Support → Work Engagement	0.262	0.000***
H6: Resonant leadership → Work Engagement	−0.302	0.033*
H7: Leader–Member Exchange → Work Engagement	0.481	0.000***
H8: Work Engagement → Perception of Unit Care Quality	0.209	0.000***
H9: Resonant leadership → Friends and Family Test	4.968	0.001**
H11: Work Engagement → Friends and Family Test	−2.201	0.034*
H12: Perception of Unit Care Quality → Falls	−0.771	0.023*
H13: Falls → Friends and Family Test	−1.273	0.000***

(b) Path	Effect (boot *SE*)	95% boot	
		Lower CI	Upper CCI
RES → POS →PUCQ → FALLS →FFT	−0.161 (0.115)	−0.481	−0.003
RES → POS →ENG → PUCQ →FALLS → FFT	0.079 (0.056)	0.002	0.242
RES → LMX →ENG → PUCQ →FALLS → FFT	0.140 (0.106)	0.003	0.451
RES → PUCQ →FALLS → FFT	0.463 (0.318)	0.004	1.294

Note

Bootstrap standard errors in parentheses. Bootstrap sample size = 10,000.

Abbreviations: ENG, Work Engagement; FFT, Friends and Family Test; LMX, Leader–Member Exchange; lower CI, lower confidence interval; POS, Perception of Organisation Support; QUAL, Unit Care Quality; RES, Resonant Leadership; *SE*, standard error; Unstandardized regression coefficients are reported; upper CI, upper confidence interval.

*p < 0.05; **p < 0.01; ***p < 0.001.

### 4.3 Path and mediation analysis

Path and mediation analysis identified four indirect mediated paths (Table 4b).

The first indirect effect is of Resonant Leadership on the Friends and Family Test through Perception of Organization Support, perception of unit care quality, and falls. This indirect effect is negative and statistically significant (bootstrap 95% CI = −0.481, −0.002). The remaining three statistically significant indirect effects were all positive. All indirect paths to Friends and Family Test were mediated by perception of unit care quality and falls and the patient safety and patient satisfaction association with resonant leadership is confirmed. Engagement, perception of unit care quality, and falls mediated the positive relationships among resonant leadership, Perception of Organization Support and Friends and Family Test, or Leader–Member Exchange and Friends and Family Test. In addition, three further paths were identified which were all mediated by perception of unit care quality and falls, from resonant leadership to Friends and Family Test.

## 5 DISCUSSION

This research explored the effects of resonant leadership, leader exchange relationships, and perceived organizational support on work engagement and patient outcomes. Our findings suggest that resonant leadership is a core antecedent of quality care. Resonant leadership also has a direct relationship with the socio‐emotional mutual investment social exchange resource between staff and patients. It also indicates that when resonant leadership is high, staff report higher quality care being delivered, associated with lower falls rates, and higher Friends and Family Test. Only two studies had previously investigated the relationship of resonant leadership to patient outcomes: 30‐day mortality (Cummings, et al., 2010) and reported medication errors (Squires et al., 2010).

These findings confirmed the role of work engagement as an emerging social exchange in reciprocity to perceived organization support and the quality of leader relationships. This extends the findings from other research where Perceived Organization Support and Leader–Member Exchange were antecedents of work engagement in relation to staff outcomes such as job satisfaction (Shacklock et al., 2013), team commitment (Dasgupta, 2016), and affective commitment (Brunetto et al., 2014; Dasgupta, 2016). Falls are a concrete and tangible example of social exchange resources (Cropanzano & Mitchell, 2005). This results from a greater mutual investment in the nurse–patient relationship as a result of the social exchange where the nurse provides a different level of nursing service or care and attentiveness to the patient, thereby preventing falls. Mutual investment in relationships by staff and patients creates a safer environment.

Engagement and its antecedents have positive effects on perceptions of unit care quality, falls rates and Friends and Family Test. This builds on the work of Dromey (2014) and West and Dawson (2012) which correlated large organizational‐level staff and patient experience data sets. Perceptions of unit care quality and falls are both mediators between the antecedents of resonant leadership and workplace relationships and the dependant variable, Friends and Family Test.

A strength of the current study was the use of institutional data to evaluate the quality of care being provided as the predominant approach in the literature was to investigate nurse‐sensitive indicators using nurse reported exposure to adverse events (Kutney‐Lee et al., 2009; Purdy et al., 2010; Squires et al., 2010; Wong et al., 2015). Until this research, falls in hospital using institutional data had not been related to social exchange theory or identified as important in mediated paths between resonant leadership and patient satisfaction (Friends and Family Test). Although Purdy et al. (2010) used inpatient satisfaction, there were no significant relationships identified with patient satisfaction. Our findings suggest researchers should make use of existing patient satisfaction data to investigate interventions to elevate resonant leadership and extend the understanding of patient experience. This is consistent with the view that patient satisfaction reflects care interactions and the culture and tone of organizations (Niederhauser & Wolf, 2018). A focus on resonant leadership is supported by the associations with lower falls rates and higher patient satisfaction (Friends and Family Test) suggesting leadership was not solely restricted to how people feel about their work and practice environment, but is translated to higher quality, particularly, patient satisfaction.

### 5.1 Limitations

The research was a cross‐sectional study with data collected at one period in time. It may therefore, be susceptible to prevalence‐incidence bias (Levin, 2006). The research was limited to one District Health Board in New Zealand and, therefore, the findings may not be translatable to other settings or professional contexts. The heterogeneous sample limits comparability with nurse‐specific samples.

The institutional independent variables were drawn from unit‐level data, whereas the LEON survey gathered individual‐level data. The resulting cross‐level effect limits interpretation of the findings to between‐team effect, not within‐team effect (Klein & Kozlowski, 2000); although Purdy et al. (2010) used a combination of individual‐level dependent variables in their multi‐level study. Future research is indicated to explore these relationships further.

## 6 CONCLUSION

This research aimed to explore the effects of resonant leadership, leader/member exchange relationships, and perceived organizational support on work engagement and patient outcomes, as nurses are held accountable (Francis Inquiry, 2013). The findings suggest that resonant leadership is a core antecedent of quality care and reinforce the unequivocal expectation of nurse leaders to assure quality care (Pegram et al., 2014). The influence of high‐ or low‐quality social exchanges on patient outcomes in highly relational contexts such as acute inpatient settings is a significant finding.

Our findings identify modifiable factors to improve staff experience of work, the safety of patient care, and ultimately patient satisfaction with their care. Work engagement mediates the relationships among resonant leadership, Perceived Organization Support and Leader–Member Exchange (separate paths), and nurse perception of unit care quality, patient outcomes (falls), and patient satisfaction (Friends and Family Test). Resonant leadership is the starting point to improve patient outcomes and has a direct effect on both perceptions of unit care quality and Friends and Family Test. All positive indirect paths to Friends and Family Test were mediated by perceptions of unit care quality and falls rates and supports the patient safety and patient experience impact of resonant leadership.

Our findings have confirmed the importance of social exchange relationships to achieve improved patient outcomes such as reduced falls rates and improved patient satisfaction. The social exchange relationships which emerge from leadership interactions and resulting obligations and reciprocity suggest an exchange of service to patients which improves care and mutual investment by staff and patients. The data support Perceived Organization Support and Leader–Member Exchange as antecedents of work engagement when investigating institutionally collected falls and Friends and Family Test. It is now possible to consider work engagement as a form of reciprocity and exchange resource. Staff engagement has been treated as a panacea for improved quality outcomes in public health systems. Our findings suggest that while engagement is important, it is not always required to provide improved experiences at work and improved patient outcomes. Rather, high‐quality relationships both with the organization and the leader are required.

The focus for nurse leaders can now shift from measuring staff engagement, to measuring patient outcomes and fostering and developing resonant leadership in practice. Indicators should be introduced which are evidenced to reveal insights into the impact of leadership on quality care, particularly falls and the Friends and Family Test. Further emphasis is required in health settings to reframe staff surveys to include social exchange components of staff experience such as perceived organization support and quality of leader–member relationships.

## CONFLICTS OF INTEREST

No conflict of interest has been declared by the author(s).

## AUTHORS’ CONTRIBUTIONS

JP undertook this project in partial fulfilment of a Doctorate of Health Sciences. JKM and ST were supervisors of the DHSc. ST provided statistical advice and guidance. All authors read and commented on major drafts and signed off the final manuscript.

### Peer Review

The peer review history for this article is available at https://publons.com/publon/10.1111/jan.14583.
